# Liquid PRF Reduces the Inflammatory Response and Osteoclastogenesis in Murine Macrophages

**DOI:** 10.3389/fimmu.2021.636427

**Published:** 2021-04-09

**Authors:** Zahra Kargarpour, Jila Nasirzade, Layla Panahipour, Richard J. Miron, Reinhard Gruber

**Affiliations:** ^1^Department of Oral Biology, Medical University of Vienna, Vienna, Austria; ^2^Department of Periodontology, School of Dental Medicine, University of Bern, Bern, Switzerland

**Keywords:** platelet-rich fibrin, inflammation, lipopolysaccharides, lactoferrin, Toll-like receptors, hemoglobin, osteoclastogenesis, osteoimmunology

## Abstract

Macrophage activation and osteoclastogenesis are hallmarks of inflammatory osteolysis and may be targeted by the local application of liquid platelet-rich fibrin (PRF). Liquid PRF is produced by a hard spin of blood in the absence of clot activators and anticoagulants, thereby generating an upper platelet-poor plasma (PPP) layer, a cell-rich buffy coat layer (BC; termed concentrated-PRF or C-PRF), and the remaining red clot (RC) layer. Heating PPP has been shown to generate an albumin gel (Alb-gel) that when mixed back with C-PRF generates Alb-PRF having extended working properties when implanted *in vivo*. Evidence has demonstrated that traditional solid PRF holds a potent anti-inflammatory capacity and reduces osteoclastogenesis. Whether liquid PRF is capable of also suppressing an inflammatory response and the formation of osteoclasts remains open. In the present study, RAW 264.7 and primary macrophages were exposed to lipopolysaccharides (LPS), lactoferrin, and agonists of Toll-like receptors (TLR3 and TLR7) in the presence or absence of lysates prepared by freeze-thawing of liquid PPP, BC, Alb-gel, and RC. For osteoclastogenesis, primary macrophages were exposed to receptor activator of nuclear factor kappa B ligand (RANKL), macrophage colony-stimulating factor (M-CSF), and human transforming growth factor-β1 (TGF-β1) in the presence or absence of PPP, BC, Alb-gel, RC lysates and hemoglobin. We show here that it is mainly the lysates prepared from PPP and BC that consistently reduced the agonist-induced expression of interleukin 6 (IL6) and cyclooxygenase-2 (COX2) in macrophages, as determined by RT-PCR and immunoassay. With respect to osteoclastogenesis, lysates from PPP and BC but also from RC, similar to hemoglobin, reduced the expression of osteoclast marker genes tartrate-resistant acid phosphatase (TRAP) and cathepsin K, as well as TRAP histochemical staining. These findings suggest that liquid PRF holds a potent *in vitro* heat-sensitive anti-inflammatory activity in macrophages that goes along with an inhibition of osteoclastogenesis.

## Introduction

Osteoimmunology was coined at the edge of the millennium when lymphocytes were discovered to drive the differentiation of hematopoietic progenitors into bone-resorbing osteoclasts (1). This process essentially depends on the activation of the RANKL-RANK signaling cascade that ultimately culminates in the expression of the genes not only characteristic of osteoclast, but also dictating their function; which is the tartrate-resistant acid phosphatase (TRAP) and the cathepsin K, removing phosphate from their substrate and the digestion of collagen of the previously solubilized mineralized extracellular matrix, respectively (2). Osteoimmunology not only provides insights into how the delicate balance of bone turnover and its shifting towards a pathological bone loss in osteoporosis and other metabolic disorders is regulated (3), but also the cross-talk of the cells representing the immune system with bone cells. Particularly, the osteoclasts was discovered to be responsible for the catabolic changes of the inflammation-induced bone loss, what we consider today as inflammatory osteolysis, a pathological process that is of significant importance in periodontitis (4, 5) and rheumatoid arthritis (6, 7).

Inflammatory osteolysis is pharmacologically targeted at the level of cytokines to interrupt the link between the activated macrophages and other immune cells to produce the inflammatory mediators, which in turn stimulate the RANKL-dependent osteoclastogenesis (8). While, rheumatoid arthritis is successfully treated with biologicals, including blocking of the major inflammatory cytokines TNFα and IL6, causing a reduction of the inflammatory symptoms (8, 9), this systemic therapeutically strategy is not feasible in oral diseases such as chronic periodontitis; an inflammation of the periodontal tissue caused by the virulence factors of the biofilm and other local mediators (4, 10). Chronic periodontitis therefore requires local therapeutic strategies, in concert with removing of the bacterial biofilm and calculus by scaling and root planning (11), to target the chronic inflammation in periodontitis with the overall aim to diminish the activation of inflammatory macrophages by their virulence factors such as LPS, and thereby reduce the formation and activation of osteoclasts.

Solid platelet-rich fibrin (PRF), which is basically the coagulated plasma-rich fraction of blood upon centrifugation, was introduced as a local therapy to support wound healing and potentially also tissue regeneration in oral indications including treatment of gingival recessions (12), increasing the width of keratinized mucosa around implants (13), to prevent atrophic bone resorption following tooth extraction (14), and to lower the symptoms that come along with inflammation such as pain and swelling upon third molar surgery (15). Support for the beneficial clinical performance of PRF application comes from *in vitro* studies showing that lysates of PRF membranes are capable of reducing the LPS-induced inflammatory response of macrophages indicated by an M1-to-M2 shift of IL6 and arginase 1 expression, respectively (16, 17), and also reduce the formation of osteoclasts in murine bone marrow cultures shown by histochemical staining of TRAP and the reduced expression of the respective gene and cathepsin K (18). Considering that both, macrophage activation (19) and osteoclastogenesis (20) require activation of the NFκB signaling pathway, and that both cells originate from the same hematopoietic progenitors (21), it might be possible that solid PRF, by reducing NFκB signaling, directly affects macrophage polarization and osteoclastogenesis. Similar data for liquid PRF are not available thus far.

Liquid PRF obtained by high-speed fractionation of blood without clot activators was termed concentrated PRF (C-PRF) (22), in contrast to injectable PRF prepared with low-speed and short-time protocols using clot activator tubes (23). Centrifugation separates the blood in a large liquid platelet-poor plasma (PPP) layer that is almost devoid of cells and the buffy coat layer accumulating the platelets and leucocytes termed concentrated PRF or C-PRF (22). It is particularly the C-PRF that is utilized to support tissue regeneration (22) and therefore used to reconstitute albumin gels (Alb-gel) prepared from heated PPP (24). The Alb-PRF gel (combination of PPP and Alb-gel) maintains volume stability for at least 4 months (25). The clinical applications of liquid PRF are thus manifold and include the production of transplantable conglomerates of fragmented solid PRF, autografts and liquid PRF (26), direct injection into a soft tissue, for example in facial esthetics (27), or to produce a growth factor-enriched matrix, exemplified by Alb-PRF (24). The impact of liquid PRF on the cellular aspects related to inhibition of inflammation and osteoclastogenesis have not been determined.

There is a rational to investigate the possible inflammatory activity of liquid PRF, as reported by us for solid PRF in a macrophage setting (16, 17) and on osteoclastogenesis (18). However, considering that the previous research on solid PRF has not considered the fractions (16–18), it remains unclear if liquid PRF produced at high speed in non-ridged (pull cap) plastic tubes exerts an anti-inflammatory activity and reduction in osteoclastogenesis, and if yes, which fraction (PPP and BC, the latter being a synonym for C-PRF) is responsible for this effect. Moreover, it is relevant to determine the possible anti-inflammatory and osteoclastogenic activity of the Alb-gel prepared from heated PPP and the remaining red clot. Here we provide data demonstrating that lysates prepared from PPP and BC exert an anti-inflammatory and together with lysates of the red clot also a reduction of osteoclastogenesis.

## Materials and Methods

### Isolation and Culture of Murine Bone Marrow‐Derived Macrophages and RAW 264.7 Cells

RAW 264.7 macrophage-like cells (LGC Standards, Wesel, Germany) were expanded in growth Dulbecco’s Modified Eagle Medium (DMEM, Sigma Aldrich, St. Louis, MO, USA), 10% fetal calf serum (Bio&Sell GmbH, Nuremberg, Germany) and 1% antibiotics (Sigma Aldrich, St. Louis, MO, USA) and seeded at 2 × 10^5^ cells/cm^2^ into 24-well plates. Primary bone marrow cells were collected from the femora and tibiae of BALB/c mice, 6- to 8-weeks (Animal Research Laboratories, Himberg, Austria). Bone marrow cells were seeded at 4 × 10^6^ cells/cm^2^ into 24-well plates and grown for 5 days in DMEM supplemented with 10% fetal bovine serum, antibiotics and with 20 ng/mL macrophage colony-stimulating factor (M-CSF; ProSpec-Tany TechnoGene Ltd, Ness‐Ziona, Israel). Bone marrow macrophages and RAW 264.7 were exposed to the respective treatments for another 24 hours under standard conditions at 37°C, 5% CO_2_, and 95% humidity. The cells were treated with LPS from *Escherichia coli* 0111: B41 (Sigma Aldrich, St. Louis, MO) at 100 ng/mL, lactoferrin at 50 ng/mL, poly (1:C) HMW (InvivoGen, Toulouse, France) at 10 µg/mL, imiquimod (InvivoGen, Toulouse, France) at 5 µg/mL, recombinant mouse IL4 (ProSpec, Ness Ziona, Israel) at 120 ng/mL in the respective experiments. All the fractions PPP, BC, Alb-gel and RC and serum were added to the cells at 10% (v/v). After overnight incubation, expression changes of respective genes were determined.

### Preparation of PPP, Buffy Coat, and Red Clot

Volunteers signed informed consent and the ethics committee of the Medical University of Vienna (1644/2018) approved the preparation of PRF. For preparing PRF gels, venous blood was collected from healthy volunteers, three females and three males from 23 to 35 years, in non-ridged (pull cap) plastic tubes (“No Additive”, Greiner Bio-One GmbH, Kremsmünster, Austria) and centrifuged at 2000 g for 8 min (swing-out rotor; Z 306 Hermle, Universal Centrifuge, Wehingen, Germany). The uppermost 2 mL PPP, the 1 mL buffy coat (BC or C-PRF), and the 1 mL erythrocyte fraction (RC) were collected. To generate Alb-gels, PPP was immediately heated at 75°C for 10 min (Eppendorf, Thermomixer F1.5, Hamburg, Germany) before being placed on ice. Every 1-ml fraction of the solid Alb-gel was then transferred into 1 ml of serum-free media. For preparation of liquid serum obtained from solid PRF, blood samples were collected into plain glass tubes (Bio-PRF, Venice, FL, USA) and centrifuged at 2000 g for 8 minutes. The solid PRF clot was squeezed out by pressing plate (Bio-PRF, Venice, FL, USA) to generate serum. The remaining PRF membrane was then divided into two parts, equivalent to PPP and BC. All fractions and the Alb-gel were subjected to dual freeze-thawing at -80°C and room temperature, respectively, followed by sonication (Sonopuls 2000.2, Bandelin electronic, Berlin, Germany) for 30 seconds. After centrifugation (Eppendorf, Hamburg, Germany) at 15,000 g for 10 min, aliquots of lysates were stored at −20°C for not longer than one month. The lysates were thawed and cells were exposed as indicated above.

### Growing Osteoclasts in Bone Marrow Cultures

Extracted bone marrow cells from femora and tibia of mice were seeded at 4 × 10^6^ cells/cm^2^ into 48-well plates and grown for 5 days in Minimum Essential Medium Eagle-Alpha Modification (αMEM) supplemented with 10% fetal calf serum (FCS) and 1% antibiotic. Receptor activator of nuclear factor kappa B ligand (ProSpec, Ness-Ziona, Israel) at 30 ng/mL, M-CSF (Cell Signaling Technology Europe, B.V., Frankfurt am Main, Germany) at 20 ng/mL, and TGF-β1 (Cell Signaling Technology Europe, B.V., Frankfurt am Main, Germany) at 10 ng/mL were used to induce osteoclastogenesis. If not otherwise indicated, 10% of PPP, BC, Alb-gel and RC or hemoglobin (Sigma Aldrich, St. Louis, MO) at 20 mM were used in the culture medium. After 6 days, histochemical staining for TRAP was performed following the instructions of the manufacturer (Sigma Aldrich, St. Louis, MO). Images were captured under a light microscope with 10X magnification (Echorevolve microscope, Euromex, Arnheim, Netherlands).

### Reverse Transcription Quantitative Real-Time PCR (RT-qPCR) and Immunoassay

For RT-qPCR (28), after overnight stimulation total RNA was isolated with the ExtractMe total RNA kit (Blirt S.A., Gdańsk, Poland) followed by reverse transcription (LabQ, Labconsulting, Vienna, Austria) and polymerase chain reaction (LabQ, Labconsulting, Vienna, Austria) on a CFX Connect™ Real-Time PCR Detection System (Bio-Rad Laboratories, Hercules, CA). Primer sequences were mIL6‐F: GCTACCAAACTGGATATAATCAGGA, mIL6‐R: CCAGGTAGCTATGGTACTCCAGAA; mCOX2-F: CAGACAACATAAAACTGCGCCTT, mCOX2-R: GATACACCTCTCCACCAATGACC; mCXCL2-F: CATCCAGAGCTTGAGTGTGACG, mCXCL2-R: GGCTTCAGGGTCAAGGCAAAC; mCCL2-F: GCTACAAGAGGATCACCAGCAG, mCCL2-R: GTCTGGACCCATTCCTTCTTGG; mCCL5-F: CCTGCTGCTTTGCCTACCTC, mCCL5-R: ACACACTTGGCGGTTCCTTC; mGAPDH‐F: AACTTTGGCATTGTGGAAGG, mGAPDH‐R: GGATGCAGGGATGATGTTCT; mARG1‐F: GAATCTGCATGGGCAACC, mARG1‐R: GAATCCTGGTACATCTGGGAAC; mYM1‐F: CATTCCAAGGCTGCTACTCA, mYM1‐R: TCATGACCTGAATATAGTCGAGAGA; mcathepsin K-F: TGTATAACGCCACGGCAAA, mcathepsin K-R: GGTTCACATTATCACGGTCACA; m TRAP-F: AAGCGCAAACGGTAGTAAGG, mTRAP-R: CGTCTCTGCACAGATTGCAT. The mRNA levels were calculated by normalizing to the housekeeping gene GAPDH using the ΔΔCt method. Supernatants were analyzed for IL6 secretion by immunoassay according to the manufacturer’s instruction (R&D Systems, Minneapolis, MN). RT-PCR data are represented compared to the untreated control, which was considered 1.0 in all the measurements so there was no need to show it as a separate group. However, in IL6 ELISA the absolute amount of secreted protein (ρg/mL) from the cells were reported, so untreated cells were also considered to show the amount of protein in all the samples and compare the protein concentration.

### Immunofluorescence

The immunofluorescent analysis of p65 nuclear translocation were performed in RAW 264.7 cells plated onto Millicell^®^ EZ slides (Merck KGaA, Darmstadt, Germany) at 15,000 cells/cm^2^. Cells were exposed to 10% of PPP, BC, Alb-gel and RC for 1 hour following overnight serum starvation. Thereafter the cells were exposed to LPS from *Escherichia coli* 0111: B41 (Sigma Aldrich, St. Louis, MO) for another 1 hour. The cells were fixed with 4% paraformaldehyde, blocked with 1% bovine serum albumin (Sigma Aldrich, St. Louis, MO) and permeabilized with 0.3% TritonX-100 (Sigma Aldrich, St. Louis, MO). We used NF-κB p65 antibodies (IgG, 1:800, Cell Signaling Technology, #8242), at 4°C overnight. Detection was with the goat anti-rabbit Alexa 488 secondary antibody (1:1000, Cell Signaling Technology, #4412). Images were captured under a fluorescent microscope with a single filter block 455 nm (Oxion fluorescence, Euromex, Arnheim, Netherlands).

### Western Blot

RAW 264.7 cells were seeded at 50,000 cells/cm^2^ into 6-well plates. The following day cells were exposed to 10% of PPP, BC, Alb-gel and RC for 30 minutes and then they were exposed to LPS for 40 minutes. Extracts containing SDS buffer with protease and phosphatase inhibitors (cOmplete ULTRA Tablets and PhosSTOP; Roche, Mannheim, Germany) were separated by SDS-PAGE and transferred onto PVDF membranes (Roche Diagnostics, Mannheim, Germany). Membranes were blocked and the binding of the first antibody NF-κB p65 antibodies (IgG, 1:1000, Cell Signaling Technology, #8242), phospho-p65 antibody (IgG, 1:1000, Cell Signaling Technology, #3031), was detected with the second antibody labelled with HRP anti-rabbit (IgG, 1:10,000, Cell Signaling Technology, #7074). After exposure to the Clarity Western ECL Substrate (Bio-Rad Laboratories, Inc., Hercules, CA) chemiluminescence signals were visualized with the ChemiDoc imaging system (Bio-Rad Laboratories). For blot densitometric analysis images were analyzed using Image Lab software (Bio-Rad Laboratories).

### Statistical Analysis

All experiments were performed four times. Every single data point is representative for an independent experiment, which is individually obtained from a different blood donor in the treatment groups. Statistical analysis of the IL6 expression and immunoassay was performed with Friedman test for multiple comparison and paired t test for single comparison. All the groups were compared with LPS, lactoferrin, poly (1:C) HMW, imiquimod, or MRT group as the positive control in the respective experiments. Analyses were performed using Prism v8 (GraphPad Software, La Jolla, CA). Significance was set at p < 0.05.

## Results

### The Anti-inflammatory Effect of PPP and BC Is Dose-Dependent in RAW 264.7 Cells

**Figure 1:**
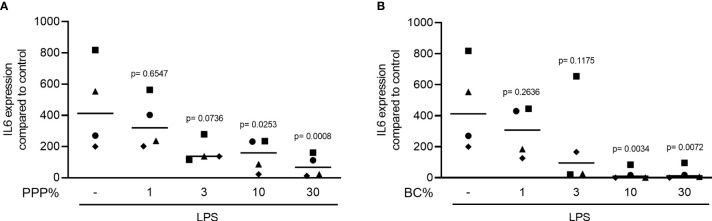
The anti-inflammatory effect of PPP and BC is dose-dependent. RAW 264.7 cells were incubated with various concentrations of PPP and BC in the presence of LPS. **(A, B)** Data represent the x-fold changes in IL6 gene expression compared to untreated control, N = 4. Statistical analysis was based on Friedman test, and P values are indicated compared to the LPS group. Significance was set at p < 0.05.

To determine the most appropriate experimental concentration of PPP and BC, we evaluated the effect of various concentrations of both fractions (0, 1, 3, 10, and 30%) on IL6 gene expression. RAW 264.7 macrophage cells were exposed to 100 ng/mL LPS either with or without aforementioned concentrations of PPP and BC. Dose-response curves revealed that concentrations above 10% could significantly suppress the LPS-induced inflammation indicated by IL6 gene expression (**Figures 1A, B**). MTT results for RC indicated that concentrations above 10% of RC were cytotoxic (data not shown). The RC fraction at 10% had no effect on the basal expression of inflammatory marker genes including IL6 and COX2 in RAW264.7 cells (data not shown).

### PPP and BC Can Suppress LPS-Induced Inflammatory Effect in RAW 264.7 Cells

**Figure 2:**
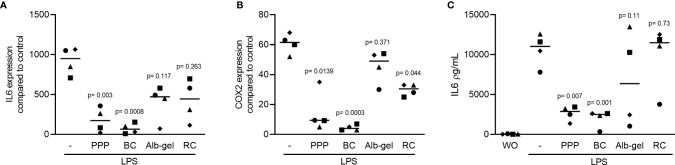
Liquid PRF fractions PPP and BC can reduce LPS-induced inflammation. RAW 264.7 cells were exposed to 10% PPP, BC, Alb-gel and RC fractions in the presence of 100 ng/mL LPS. **(A, B)** Data show the x-fold changes of IL6 and COX2 gene expression **(C)** and the IL6 levels in the cell supernatant, N = 4. Statistical analysis was based on Friedman test, and P values are indicated compared to the LPS group. Significance was set at p < 0.05.

**Figure 3:**
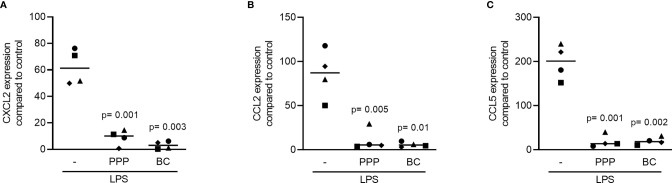
Liquid PRF fractions PPP and BC can suppress LPS-induced chemokines. RAW 264.7 cells were treated with 10% PPP and BC fractions in the presence of 100 ng/mL LPS to evaluate expression changes of LPS-induced chemokines. **(A–C)** Data show the x-fold changes of CXCL2, CCL2 and CCL5 in the presence of PPP and BC, N = 4. Statistical analysis was based on Friedman test, and P values are indicated compared to the LPS group. Significance was set at p < 0.05.

To evaluate the anti-inflammatory effect of different fractions of liquid PRF (22, 26), the RAW 264.7 macrophage cell line was exposed to LPS either without or with 10% of PPP, BC, Alb-gel and RC. Gene expression analysis showed that PPP and BC fractions are both able to notably reduce the LPS-induced inflammatory effects. Alb-gel and RC fractions, however, failed to significantly reduce expression of IL6 and COX2 (**Figures 2A, B**). To confirm these findings obtained by gene expression analysis, we measured the levels of IL6 protein in the supernatant of RAW264.7 cells. Consistently, PPP and BC, but not Alb-gel and RC, reduced the production of IL6 (**Figure 2C**). To additionally approve the anti-inflammatory effect of PPP and BC, we measured the expression of LPS-induced chemokines CXCL2, CCL2 and CCL5 in the RAW 264.7 cells. Consistently, expression of all chemokines was significantly suppressed by PPP and BC (**Figures 3A–C**).

### PPP and BC Can Reduce Lactoferrin and TLR Agonist-Induced Inflammation in RAW 264.7 Cells

**Figure 4:**
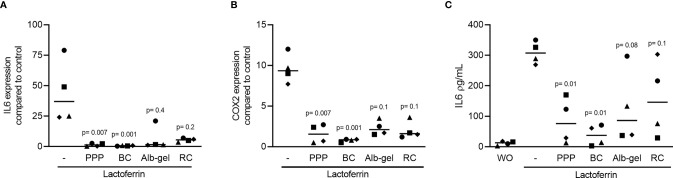
PPP and BC can suppress lactoferrin-induced inflammation in RAW 264.7. The cells were exposed to 10% PPP, BC, Alb-gel and RC fractions in the presence of 50 μg/mL lactoferrin. **(A, B)** Data show the x-fold changes of IL6 and COX2 gene expression and **(C)** the IL6 levels in the cell supernatant, N = 4. Statistical analysis was based on Friedman test, and P values are indicated compared to the lactoferrin group. Significance was set at p < 0.05. WO means without and represents unstimulated cells.

**Figure 5:**
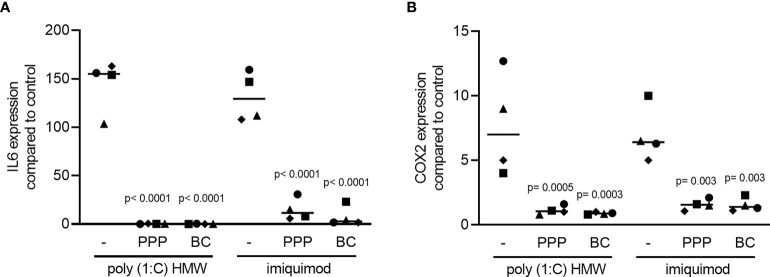
PPP and BC can significantly reduce inflammation induced by TLR agonists. The cells were exposed to 10% PPP, BC in the presence of 10 μg/mL poly (1:C) HMW and 5 μg/mL imiquimod, agonists of TLR3 and TLR7 respectively. **(A, B)** Data show the x-fold changes of IL6 and COX2 gene expression, N = 4. Statistical analysis was based on Friedman test, and P values are indicated compared to the TLR agonists group. Significance was set at p < 0.05.

To understand if the anti-inflammatory activity of PPP and BC is limited to LPS, and considering that LPS is possibly scavenged by the HDL, we have generated an inflammatory response in RAW 264.7 by lactoferrin (29). We report here that in the presence of lactoferrin, all the fractions including PPP, BC, Alb-gel and RC are able to reduce the expression of IL6 and COX2 in RAW 264.7, but only PPP and BC could reach the significant level of reduction (**Figures 4A, B**). In support with the gene expression data, PPP and BC greatly reduced the IL6 protein levels provoked by lactoferrin in the macrophage cell line (**Figure 4C**). Furthermore, RAW264.7 cells were treated with poly (1: C) HMW and imiquimod, agonist of TLR3 and TLR7, respectively, and also in this setting, PPP and BC significantly reduced the expression of IL6 and COX2 (**Figures 5A, B**).

### Serum From Solid PRF Can Reduce LPS-Induced Inflammation in RAW 264.7 Cells

**Figure 6:**
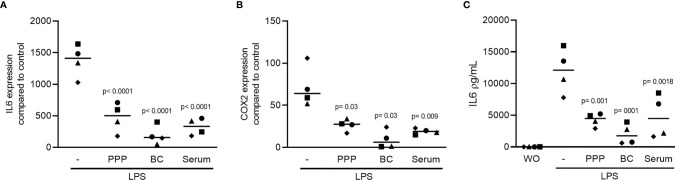
The solid PPP, solid BC and serum are able to reduce LPS-induced inflammation. To confirm that also the serum produced from solid PRF hold an anti-inflammatory activity, jelly solid PRF was squeezed out to collect the PRF serum. RAW 264.7 cells were exposed to 10% solid PPP, solid BC, and serum in the presence of 100 ng/mL LPS. **(A, B)** Data show the x-fold changes of IL6 and COX2 gene expression and **(C)** the IL6 levels in the cell supernatant, N = 4. Statistical analysis was based on Friedman test, and P values are indicated compared to the LPS group. WO means without and represents unstimulated cells.

To see if the serum produced from solid PRF (30) also holds an anti-inflammatory activity, solid PRF clots was squeezed out to collect the serum. The remaining PRF membrane was cut into a PPP and a BC part and lysates were prepared as described recently (17). RAW 264.7 macrophage cell line was exposed to 100 ng/mL LPS either without or with 10% of the solid PPP, solid BC and serum. All the fractions substantially lowered LPS-induced inflammation indicated by the expression of IL6 and COX2 (**Figures 6A, B**). To confirm the findings obtained by gene expression analysis, we measured accumulation of IL6 protein in the supernatant of RAW264.7 cells (**Figure 6C**).

### PPP and BC Can Suppress Phosphorylation and Nuclear Translocation of p65 in RAW 264.7

**Figure 7:**
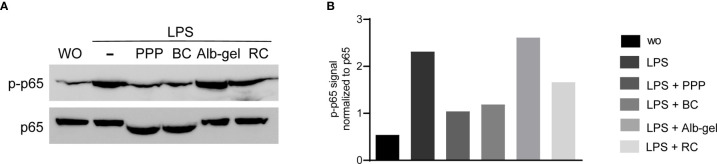
PPP and BC layers can suppress phosphorylation of p65 in RAW 264.7. Western blot analysis was carried out for phospho-p65 and total p65. **(A)** Raw cells were treated with LPS in the presence or absence of 10% of PPP, BC, Alb-gel and RC. **(B)** Data indicate the relative changes normalized to p65 and unstimulated control cells.

**Figure 8:**
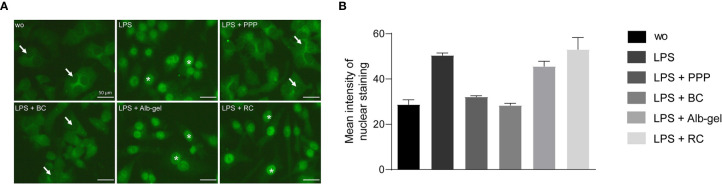
PPP and BC attenuate the translocation of NFkB from the cytoplasm into the nucleus. Raw 264.7 cells were exposed to LPS with or without PPP, BC, Albgel and RC. **(A)** Immunofluorescence analysis of intracellular translocation of NFkB p65 into the nucleus, induced by LPS in RAW 264.7. WO means without and represents unstimulated cells. Stars represent stained nuclei and arrows represent non-stained nuclei for more clarity. **(B)** Data indicate the mean signal intensity of nuclear staining by obtained by ImageJ software.

To confirm the anti-inflammatory activity of PPP and BC, Western blot analysis was carried out for phospho-p65. PPP and BC suppressed the phosphorylation of p65 and even caused a gel shift of p65 in RAW 264.7 (**Figure 7A**) being quantified by densitometry (**Figure 7B**). To further evaluate the inhibitory effect of PPP and BC on NF-κB signaling, we performed an immunofluorescent analysis of NF-κB nuclear translocation. LPS caused a clear p65 nuclear staining that was however not observed in the presence of PPP and BC (**Figure 8A**). Histogram analysis of the signal intensity support the visual impression of the nuclear staining (**Figure 8B**). Thus, the anti-inflammatory activity of PPP and BC is associated with a reduction of p65 phosphorylation and its nuclear translocation.

### PPP and BC Can Provoke an M1-to-M2 Polarization Shift in Murine Bone Marrow Cells

**Figure 9:**
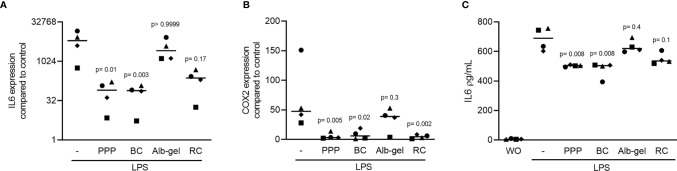
Liquid PRF fractions PPP and BC suppress LPS-induced inflammation in macrophages. Bone marrow macrophages were exposed to 10% PPP, BC, Alb-gel and RC fractions in the presence of 100 ng/mL LPS. **(A, B)** Data show the x-fold changes of IL6 and COX2 gene expression and **(C)** the IL6 levels in the cell supernatant, N = 4. Statistical analysis was based on Friedman test, and P values are indicated compared to the LPS group. WO means without and represents unstimulated cells.

**Figure 10:**
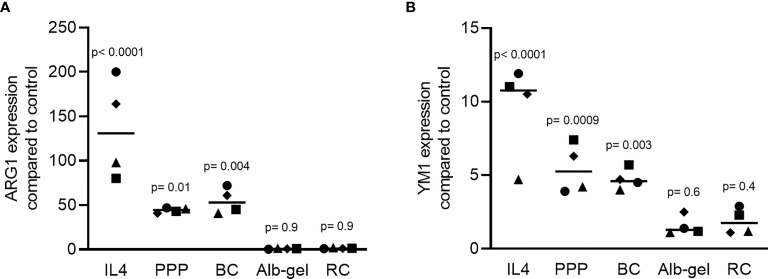
PPP and BC but not Alb-gel and RC induce the formation of M2 pro-resolving macrophages. Bone marrow macrophages were exposed to 120 ng/mL IL4 or 10% PPP, BC, Alb-gel and RC. **(A–B)** Data show the x-fold changes of ARG1 and YM1 expression in bone marrow cultures; N = 4. Statistical analysis was based on Friedman test, and P values are indicated comparing mean of each group with the mean of untreated control group.

To confirm the findings observed in the RAW 264.7 cells, primary macrophages from murine bone marrow were similarly exposed to LPS in the presence or absence of 10% PPP, BC, Alb-gel and RC. Consistently, PPP and BC but not Alb-gel and RC strongly suppressed the LPS-provoked inflammation in primary macrophages indicated by the reduced expression of IL6 (**Figure 9A**). Interestingly all the fractions but Alb-gel could significantly reduce expression of COX2 (**Figure 9B**). In line with the gene expression data, PPP and BC but not Alb-gel and RC substantially reduced the IL6 protein levels induced by LPS the supernatant of primary macrophages (**Figure 9C**). Moreover, as with IL4 (31), PPP and BC induced an M2 macrophage polarization indicated by robust expression of arginase 1 (ARG1; **Figure 10A**) and chitinase-like protein 3 (Chil3; YM1; **Figure 10B**). Thus, PPP and BC provoke a macrophage polarization from pro-inflammatory M1 toward pro-resolving M2 phenotypes that can, for example, suppress osteoclast differentiation in a periodontitis model (32).

### Osteoclast Formation Was Suppressed in the Presence of PPP, BC and RC but Not Alb-Gel

**Figure 11:**
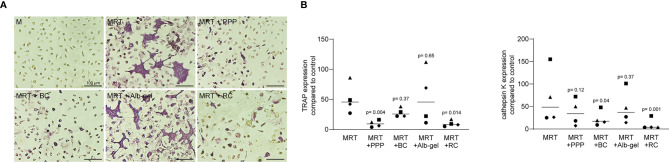
Osteoclast formation is suppressed in the presence of PPP, BC and RC. Murine Bone marrow cells were grown in the presence of 10% PPP, BC, Alb-gel and RC to modify osteoclastogenesis induced by M‐CSF, RANKL and TGF‐β (MRT). **(A)** Representative images of TRAP^+^ multinucleated osteoclasts in the control group M-CSF (M) and in the absence or presence of PPP, BC, Alb-gel and RC. **(B)** Data represent the x‐fold changes in expression of osteoclast marker genes, TRAP and cathepsin K compared to an M‐CSF control, N=4. Statistical analysis was based on Friedman test.

**Figure 12:**
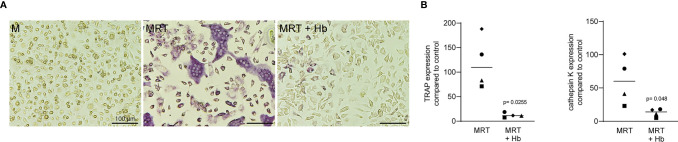
Osteoclast formation is suppressed in the presence of hemoglobin. Murine Bone marrow cells were grown in the presence of 20 mM hemoglobin (Hb) to modify osteoclastogenesis induced by M-CSF, RANKL and TGF-β (MRT). **(A)** Representative images of TRAP^+^ multinucleated osteoclasts in the control group M-CSF (M) and in the absence or presence of Hb. **(B)** Data represent the x-fold changes in expression of osteoclast marker genes, TRAP and cathepsin K compared to an M-CSF control, N=4. Statistical analysis was based on Paired t test.

We then assessed the direct role of liquid PRF fractions in osteoclast differentiation. Murine bone marrow cells cultivated by M‐CSF, RANKL and TGF-β (MRT) were treated with or without 10% of PPP, BC, Alb-gel and RC fractions. We showed that PPP, BC and RC fractions can decrease the size and number of TRAP-positive multinucleated cells using TRAP histochemical staining (**Figure 11A**). In line with this observation, PPP, BC and RC reduced the expression of TRAP and cathepsin K significantly, both enzymes required for bone resorption (**Figure 11B**). Alb-gel failed to substantially reduce the number of osteoclasts and the expression of osteoclast marker genes (**Figure 11B**). PPP, BC and RC had no impact on the expression of INFγ and IL12, both osteoclastogenesis inhibitors (data not shown). Hemoglobin also reduced osteoclast formation what may explain the findings obtained with the RC lysates (**Figures 12A, B**).

## Discussion

Liquid PRF prepared in regular plastic tubes at high-speed centrifugation (22, 26) provides the cell-rich buffy coat layer of C-PRF (22) and the platelet-poor plasma (PPP), the latter being a rich source of fibrinogen to prepare conglomerates of bone grafts (26) or albumin gels (Alb-gel) upon heating (24). It is thus the C-PRF but also the PPP that is clinically applied in regenerative dentistry. The proposed beneficial activity of liquid PRF that includes the lowering of the local inflammation and the reduction of osteoclastogenesis is supposed to be caused by the C-PRF being a rich-source of platelets and leucocytes (33), however, if also PPP being almost devoid of cells exerts an anti-inflammatory and anti-osteoclastogenic activity remains unclear. Even more enigmatic is the possible anti-inflammatory and anti-osteoclastogenic activity of the heated PPP, the Alb-gel. Here we provide some basal insights into the possible anti-inflammatory and anti-osteoclastogenic activity of the various fractions and preparations of liquid PRF based on murine bone marrow *in vitro* models.

The first main finding of the present study was that not only BC lysates have the expected anti-inflammatory activity (17), but also PPP lysates caused a robust inhibition of the inflammatory response of macrophages exposed to the classical TLR4 agonist LPS that goes along with a reduced p65 phosphorylation and nuclear translocation, overall suggesting a diminished NFκB signaling. The same is true when macrophages are stimulated with another TLR4 agonist namely lactoferrin (34), and poly (1:C) HMW and imiquimod, agonists of TLR3 and TLR7, respectively. Thus, the inhibition of inflammation caused by the PRF lysates is not specific for any TLRs. Moreover, the anti-inflammatory activity reported herein, indicated by the cytokine IL6 and COX2 the key enzyme of prostaglandin E_2_ production, was also shown for a chemokine panel, CXCL2, CCL2 and CCL5.

The second main finding was that lysates of BC and PPP induce an M1-to-M2 polarization switch. For that, the anti-inflammatory activity BC and PPP was confirmed in macrophages generated from murine bone marrow. In extension of this information, we investigated the potential that the macrophages are not only suppressed in their M1-inflammatory status but shifted towards the M2-resolving status. Indeed, lysates of BC and PPP increased the expression of ARG1 and YM1, both being markers for M2 macrophages. These findings support our previous observation that lysates of solid PRF cause an M1-to-M2 polarization switch (17), and are partially consistent with PPP prepared with an apheresis system, where PPP reduced nitric oxide but not TNFα and COX2 expression of LPS-stimulated RAW 264.7 macrophages (33). Thus, our data suggest that it is not only the cell-rich BC but also the PPP that holds a potent anti-inflammatory activity that extends towards an M2-polarisation of macrophages in murine bone marrow cultures.

The third main finding of the current research was that lysates of the BC thus C-PRF and PPP reduced the formation of osteoclasts *in vitro* – similar to what we have shown for lysates of solid PRF (18). Again, it seems that the inhibition of osteoclastogenesis is not necessity caused by platelets, but more likely by parts of the PPP that are also present in BC and consequently C-PRF. Moreover, activated platelets depleted for leucocytes and freed from plasma components support osteoclastogenesis in murine bone marrow cultures (35), and it was the serum that reduced osteoclastogenesis *in vitro* (36). However, we have not expected the robust inhibition of osteoclastogenesis by lysates of the RC. Searching for possible explanations, we show here that also hemoglobin alone can suppress osteoclastogenesis. These findings are supported by observations that oxidized ferryl hemoglobin greatly abolished osteoclastogenesis *in vitro* (37). In vivo, upon hemolysis hemoglobin is released from red blood cells to the circulation and give a rise to heme which has pro-inflammatory properties (38). Even though, hemoglobin is linked to TLR4 signaling in the epithelium (39) and TLR4 signaling suppresses osteoclastogenesis in murine bone marrow cultures (40, 41) (42), this mechanism is unlikely in our model because RC lysates failed to activate TLR4 signaling in RAW 264.7 cells.

Our findings observed with heated PPP that turns into Alb-gel are also interesting. Compared to the lysates of PPP, the lysates of Alb-gel are considerably less potent to lower the LPS and lactoferrin-induced IL6 expression. These findings suggest that the molecules responsible for the anti-inflammatory activity of PPP are heat sensitive. Nevertheless, in RAW 264.7 macrophages exposed to LPS or lactoferrin, there is a remaining anti-inflammatory activity on Alb-gel, even though not reaching a level of significance. This observation may help to identify which components in PPP, and presumably also BC, are responsible for the lowering of IL6 and COX2 expression in macrophages. There are candidate molecules such as apolipoprotein A-I of HDL to adsorb and thereby lower the LPS-induced inflammatory response (43). Also serum components such as high-density lipoprotein (44, 45) and the content of apolipoprotein A-I exert an anti-inflammatory activity by interfering with the TLR system (46). From a clinical perspective, not only the C-PRF but also PPP is useful, maybe not as a primary source of platelet-derived growth factors, but because of its osteoimmunological activity on macrophages and osteoclasts (24).

Taken together, we report here that not only lysates prepared from the cell-rich BC or C-PRF but also from the plasma PPP layer of liquid PRF exert an anti-inflammatory as well as an anti-osteoclastogenic response in macrophage cultures, presumably involving heat sensitive components lowering the NFκB signaling. Whether or not that the anti-inflammatory as well as the anti-osteoclastogenic response are mediated by the same or different plasma-derived components and pathways remains to be determined. Also understanding the possible role of the platelets and the leucocytes in this context is an inspiration for future research. The study provides another novel aspect to PRF research namely that the lysates of RC similar to hemoglobin suppressed osteoclastogenesis.

## Data Availability Statement

The original contributions presented in the study are included in the article/supplementary material. Further inquiries can be directed to the corresponding author.

## Ethics Statement

The studies involving human participants were reviewed and approved by ethics committee of the Medical University of Vienna (1644/2018). The patients/participants provided their written informed consent to participate in this study.

## Author Contributions

ZK contributed to conceptualization and design, methodology, acquisition, analysis, software, validation and interpretation, critically revised manuscript, gave final approval, agreed to be accountable for all aspects of work. JN contributed to acquisition, analysis, interpretation, critically revised manuscript, gave final approval, agreed to be accountable for all aspects of work. LP contributed to acquisition, analysis, interpretation, critically revised manuscript, gave final approval, agreed to be accountable for all aspects of work. RM critically revised manuscript, gave final approval, agreed to be accountable for all aspects of work. RG contributed to conception and design, acquisition, analysis, and interpretation, drafted manuscript, critically revised manuscript, gave final approval, agreed to be accountable for all aspects of work. All authors contributed to the article and approved the submitted version.

## Funding

This research was funded in part by a grant from the Osteology Foundation, Switzerland (17-125 and 17-219). ZK and JN received support from the Austrian Science Fund (FWF) (4072-B28).

## Conflict of Interest

The authors declare that the research was conducted in the absence of any commercial or financial relationships that could be construed as a potential conflict of interest.
